# Post-COVID-19 surge in Guillain-Barré syndrome during the Omicron wave in China with clinical characteristics and potential immune-mediated pathways

**DOI:** 10.1038/s41598-026-44136-w

**Published:** 2026-03-13

**Authors:** Jiwei Zhang, Yifan Guo, Liting Wei, Jinshun Liu, Shibo Li, Shuo Zhang

**Affiliations:** 1 Department of Neurology, The First Affiliated Hospital of Zhengzhou University, Zhengzhou, China; 2 Translational Medicine Center, The First Affiliated Hospital of Zhengzhou University, Zhengzhou, China; 3 Department of Neurology, Luoyang Central Hospital Affiliated to Zhengzhou University, Luoyang, China

**Keywords:** Guillain–Barré syndrome (GBS), COVID-19, Omicron variant, Incidence, Immune-mediated mechanism, Epidemiological surge, Diseases, Health care, Medical research, Neurology

## Abstract

This multicenter study investigated the epidemiological and clinical characteristics of Guillain-Barré syndrome (GBS) during China’s Omicron wave (December 2022–February 2023), and compared the number of GBS hospitalizations with the historical data for the same period from 2018 to 2022. A retrospective analysis was conducted at two tertiary hospitals, categorizing patients into COVID-GBS (case group) and Non-COVID-GBS (control group). During the Omicron wave, the number of GBS hospitalizations was 1.5 times higher compared to the period of 2018–2019 (99 cases vs. 66 cases). Poisson regression analysis confirmed a significant increase in GBS incidence during the Omicron wave (December 2022–February 2023) compared to the 2018–2019 baseline period, with an IRR of 1.541 (95% CI: 1.123–2.129, *p* = 0.0079). COVID-19-associated GBS patients were significantly older (54.04 vs. 42.06 years, *p* = 0.002) and exhibited higher rates of cranial nerve involvement (*p* = 0.014), particularly bulbar involvement (*p* = 0.009). Acute severity was greater in COVID-19-associated cases, evidenced by elevated ICU admissions, higher peak GBS disability scores (*p* = 0.048), increased mechanical ventilation needs, and one fatality. The median latency from COVID-19 infection to neurological onset was 9.5 days (IQR: 8–14). Despite these acute differences, 6-month disability outcomes showed no significant divergence between groups, suggesting similar long-term prognoses. The surge in GBS incidence aligns with broader reports of elevated GBS rates during COVID-19 surges, though mechanistic links may involve immune-mediated pathways rather than direct viral causation.

## Introduction

Coronavirus disease 2019 (COVID-19), caused by the severe acute respiratory syndrome coronavirus 2 (SARS-CoV-2), has become a global pandemic. Following the relaxation of COVID-19 restrictions on December 7, 2022, China experienced widespread COVID-19 infections, largely driven by the Omicron subvariants BA.5.2 and BF.7, according to data from the Chinese Center for Disease Control^1^. BA.5.2 and BF.7 together accounted for 97.5% of all local infections as per genomic sequencing^1^. Nationwide, SARS-CoV-2 infection rate peaked between December 19 and 21, 2022, with 82.4% of the Chinese population being infected as of February 7, 2023^2^. As of January 6, 2023, the COVID-19 infection rate in Henan Province was 89.0%, with 89.1% in urban areas and 88.9% in rural areas, predominantly attributed to the Omicron variant^3^.

The main manifestation of COVID-19 is interstitial pneumonia, which rapidly leads to respiratory failure^4,5^. However, with the deepening understanding of the disease and the increase in case numbers, many non-pulmonary symptoms have been recognized, including neurological complications such as acute cerebrovascular diseases, meningitis, encephalitis, and specific neuromuscular manifestations including Guillain–Barré syndrome (GBS)^6,7^.

GBS is an acute inflammatory disorder of the peripheral nerves and clinically characterized by progressive limb weakness, sensory deficits, cranial nerve involvement, tendon areflexia, and albuminocytological dissociation of cerebrospinal fluid (CSF) albumin^8,9^ and it typically follows a rapidly progressive, monophasic course, reaching its nadir within 4 weeks. It is an immune-mediated polyneuropathy often triggered by antecedent infections. Two-thirds of adult patients report preceding symptoms of respiratory or gastrointestinal tract infection in the four weeks preceding weakness onset^10^. ​Infection with *C. jejuni* is one of the most commonly identified antecedent^10^. Other associated pathogens include cytomegalovirus, Epstein-Barr virus, *Mycoplasma pneumoniae*, hepatitis B virus, human immunodeficiency virus, and Zika virus^11^. One of the most recognized mechanisms of GBS following infection is molecular mimicry^12^. The antigenic structure of some pathogens is highly similar to the components in the peripheral nerve tissue, such as gangliosides, which cause the immune system to mistakenly attack the nerve tissue^12^. Although SARS-CoV-2 primarily causes respiratory symptoms, its surface antigens, particularly the Spike protein, may trigger cross-immune responses against neural tissues through a molecular mimicry mechanism.

During the previous COVID-19 pandemic, cases of COVID-19 and GBS worldwide were described, and the increasing number of reported cases suggests a possible link between the two conditions^13,14^. However, several large-scale epidemiological studies did not confirm a significant increase in the incidence of GBS incidence at the population level^15,16^. An important consideration is that many of these studies were carried out in settings under strict lockdown policies. While such measures limited the spread of SARS-CoV-2, they also likely reduced circulation of other common triggers of GBS potentially obscuring any specific risk attributable to the virus itself. Thus, the association between COVID-19 and GBS remains incompletely defined. The Omicron pandemic in China in late 2022 created such an unprecedented “natural experiment” scenario: extremely high infection rates, large-scale population infection and no interference from lockdown and control measures. This study aimed to take advantage of this unique condition to compare the incidence of GBS hospitalization during the COVID-19 Omicron epidemic with that before the outbreak, to explore whether there is a temporal association between Omicron infection and GBS onset and to describe the unique clinical features of Omicron associated GBS and possible mechanisms.

## Methods

### Patients and methods

This retrospective study was conducted at the First Affiliated Hospital of Zhengzhou University and Luoyang Central Hospital, two tertiary hospitals. Data were collected from hospitalized patients diagnosed with GBS between December 2022 and February 2023, and case numbers were compared with those from the corresponding period (December to February) during 2018–2022 at the same institutions. Additionally, the total number of inpatients in the Department of Neurology during the same period was collected, and the ratio of GBS patients to total inpatients was calculated to assess the relative incidence frequency.

Patients hospitalized in the Department of Neurology from December 7, 2022, to February 28, 2023, with SARS-CoV-2 infection within six weeks prior to GBS onset were included in the Case Group (COVID-GBS). Patients with evidence of other triggers were excluded, including infection with other common respiratory pathogens and the presence of gastrointestinal symptoms. COVID-19 diagnosis was confirmed by reverse transcription polymerase chain reaction (RT-PCR) detection of SARS-CoV-2 RNA in nasopharyngeal swabs. However, owing to the scarcity of diagnostic tests during the pandemic outbreak, COVID-19 diagnosis was accepted based on confirmed epidemiological exposure, characteristic symptoms, and/or typical pulmonary parenchymal infiltration observed on chest CT scans. Among the 55 patients in the COVID-GBS group, ​12 patients (21.8%)​​ had their SARS-CoV-2 infection confirmed by a positive ​RT-PCR test​ on nasopharyngeal swab, which represents the gold standard for laboratory confirmation. The remaining ​43 patients (78.2%)​​ were classified based on a ​clinical and epidemiological rationale​ validated in contexts of high community transmission and they had a positive result using a commercially available COVID-19 Antigen Detection Kit. This approach is recognized as reliable during periods of intense outbreak dynamics, such as the Omicron wave in our study period (December 2022 to February 2023), when population infection rates were estimated to exceed 80%^3^. The high prevalence of the virus during this period makes recent symptomatic infection a strong and probable antecedent trigger. To accurately estimate the interval from infection to GBS onset, the date of infection was defined as follows: for patients with a positive RT-PCR test obtained via nasopharyngeal swab within 3 days after symptom onset, the date of sampling was used; for patients without a RT-PCR test but with typical clinical symptoms of COVID-19 diagnosed by a physician, the date of symptom onset was used. Patients hospitalized at the same institutions before the major COVID-19 outbreak without evidence of SARS-CoV-2 infection before or during GBS onset were assigned to the Control Group (Non-COVID-GBS).

All patients in both groups were diagnosed with newly onset GBS with neurological symptoms based on the diagnostic criteria established by the European Academy of Neurology/Peripheral Nerve Society Guidelines^17^. The diagnosis was confirmed by two neurologists in the Department of Neurology through the incorporation of clinical presentation, CSF analysis, electrophysiological studies conducted during hospitalization, and testing for ganglioside antibodies. Patients were excluded if they presented with non-acute or remission-phase GBS; chronic inflammatory demyelinating polyneuropathy; paraneoplastic syndromes; diabetic peripheral neuropathy; or alternative neuropathies such as toxic, metabolic, or hereditary types. Demographic and clinical manifestations of GBS, CSF examination, electrophysiological examination, treatment, disease progression, and prognosis were retrospectively assessed in both groups. Patients were classified according to both clinical and electrophysiological subtypes. Clinical subtyping was based on the predominant presenting symptoms and neurological signs at admission, as per established diagnostic criteria^18^. Electrophysiological subtyping^19^ was performed based on the results of nerve conduction studies (NCS) conducted within the first two weeks of symptom onset. Telephone follow-ups were conducted to evaluate long-term outcomes.

### Clinical scale

Disease severity was expressed using the GBS disability score, also known as the Hughes Disability Scale (0–6)^17,20^. This seven-point scale is as follows: 0 (no symptoms), 1 (minor symptoms and capable of running), 2 (able to walk 10 m without assistance but unable to run), 3 (able to walk 10 m across an open space with help; unable to walk unaided), 4 (unable to walk 10 m even with help; wheelchair bound or bedridden), 5 (requiring assisted ventilation for at least part of the day), and 6 (dead).

### Statistical analysis

The statistical analysis was conducted using the IBM SPSS software (version 26.0; IBM Corp., Armonk, NY, USA). Continuous variables are presented as median values and/or mean ± SD based on their distributional properties. Categorical variables are reported as frequencies and percentages. Measurement data were analyzed using either the t-test or the Mann–Whitney U-test, depending on their normality. Enumeration data were analyzed using the chi-square test. For ordinal variables, differences in the distribution of ranks between the two independent groups were analyzed using the Mann–Whitney U test. The threshold for statistical significance was set at *p* < 0.05. We used Poisson regression to compare the incidence of GBS hospitalizations during the Omicron wave (December 2022 to February 2023) with the pre-pandemic baseline period (December 2018 to February 2019). The model included the total number of neurology inpatients as an offset term to account for variations in hospital capacity. Incidence rate ratios (IRR) with 95% confidence intervals (CI) were calculated. Additionally, we performed a multivariable linear regression to assess the association between Omicron status and the 6-month GBS disability score, adjusting for age, gender, and baseline clinical features.

## Results

**Fig. 1:**
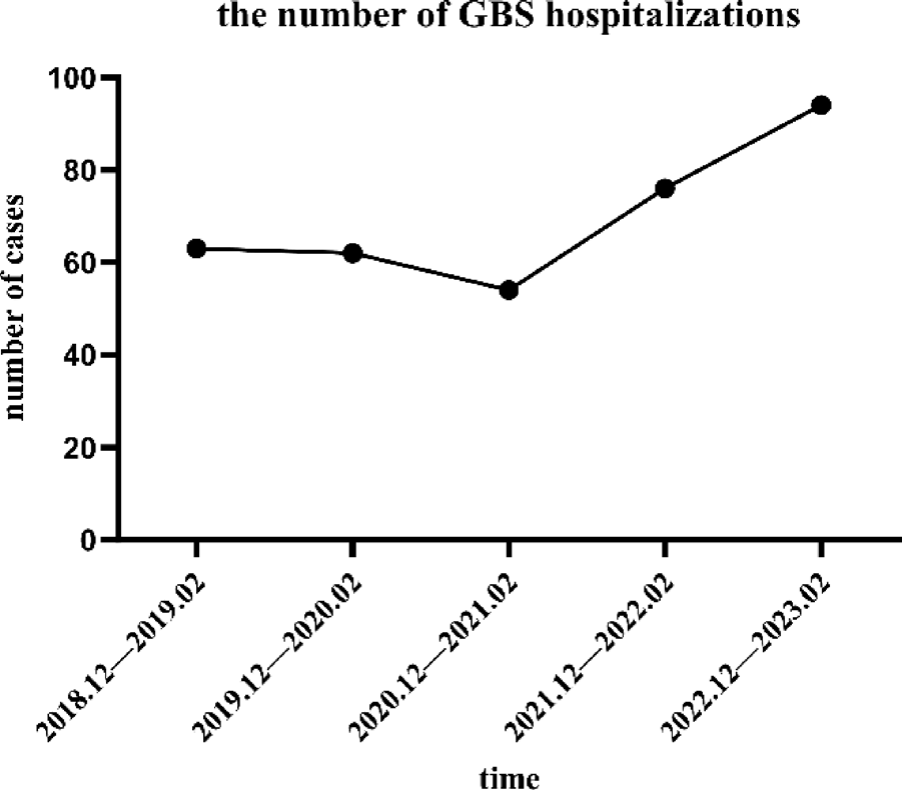
Number of hospitalizations for Guillain–Barré Syndrome (GBS) from December to February each year between 2018 and 2022.

**Fig. 2:**
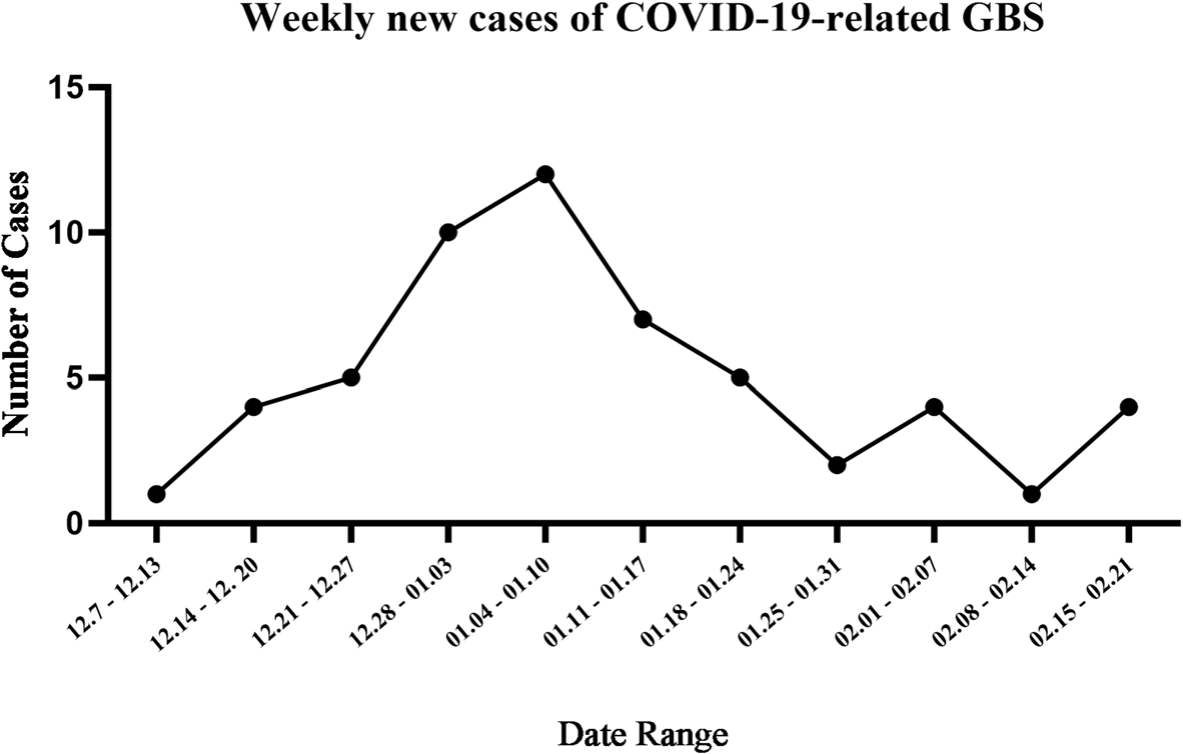
Number of new cases per week based on the onset time of GBS patients infected with SARS-CoV-2.

From December 2022 to February 2023, the number of GBS hospitalizations demonstrated an increasing trend compared to corresponding periods in previous years (Fig. 1). A total of 5 and 94 GBS inpatients were admitted at Luoyang Central Hospital and The First Affiliated Hospital of Zhengzhou University, respectively, between December 2022 and February 2023, compared to 3 and 63 cases during the same period in 2018–2019. Combined data from both hospitals showed 99 GBS cases during the Omicron period versus 66 in the pre‑pandemic control period, representing an approximately 1.5‑fold increase. The proportion of GBS admissions within the Department of Neurology at The First Affiliated Hospital increased from 1.05% (63/6000) in 2018–2019 to 1.62% (94/5810) during the Omicron wave. Poisson regression analysis confirmed a statistically significant increase in incidence during the Omicron period compared with the 2018–2019 baseline, with an IRR of 1.541 (95% CI 1.123–2.129, *p* = 0.0079), which was consistent with exact Poisson test results (IRR = 1.541, 95% CI 1.108–2.155, *p* = 0.0083). This trend suggests a significant increase in GBS incidence during the COVID-19 outbreak. Among 99 patients with GBS hospitalized between December 2022 and February 2023, 55 were classified into the COVID-GBS group. These patients had new-onset neurological manifestations after December 7, 2022, and had a current or prior SARS-CoV-2 infection within 6 weeks. A detailed review of antecedent events within the 6 weeks preceding GBS onset was conducted for all patients. In the COVID-GBS group, SARS-CoV-2 infection was identified as the antecedent trigger in all cases. The median interval from antecedent infection to GBS-related clinical manifestation onset was 9.5 days (interquartile range: 8–14 days), with limb weakness representing the most common clinical symptom. Temporal distribution analysis of disease onset by week revealed a clear incidence peak between January 4 and January 10, 2023 (Fig. 2), consistent with the 2022–2023 regional outbreak pattern. Within the Non-COVID-19 GBS control group (*n* = 48), a diverse spectrum of antecedent events was documented. The most frequently identified triggers were respiratory tract infections (18/48, 37.5%) and gastrointestinal infections (10/48, 20.83%). Other antecedent events included recent vaccination (2/48, 4.2%), surgical procedures (2/48, 4.2%), childbirth (1/48, 2.1%), and herpes zoster infection (1/48, 2.1%). No specific antecedent event was identified in 14 patients (29.17%).

**Fig. 3:**
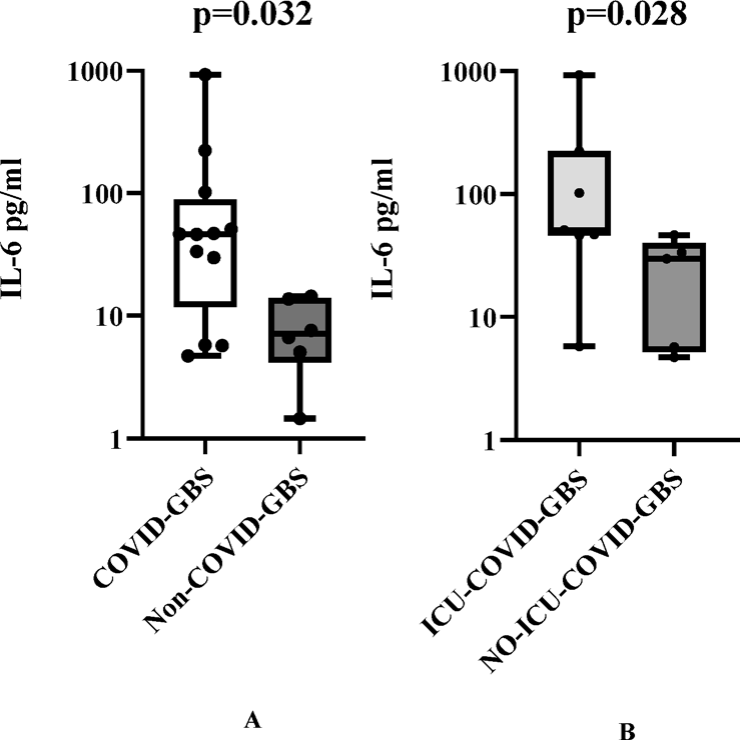
**(A)** IL-6 levels in patients with COVID-19-associated GBS vs. in those with COVID-19-negative GBS. **(B)** IL-6 levels in patients with COVID-19 requiring ICU admission vs. in those not requiring ICU.

We summarized the clinical characteristics of the total cohort and compared patients with and without SARS-CoV-2 infection (COVID-GBS and Non-COVID-GBS) (Table 1). COVID-GBS patients were significantly older than those without SARS-CoV-2 infection (54.04 ± 15.803 vs. 42.06 ± 21.147, *p* = 0.002). Both case and control groups were predominantly male (72.72% and 60.42%, respectively). Common neurological features included limb weakness, sensory disturbances, diminished or absent tendon reflexes, ataxia, and cranial nerve involvement. The COVID-GBS group demonstrated significantly greater cranial nerve involvement compared to the Non-COVID-GBS group (*p* = 0.014), particularly bulbar palsy (*p* = 0.009). Although not statistically significant, COVID-19-infected GBS patients exhibited trends toward more severe disease, evidenced by higher ventilator dependency rates (*p* = 0.059), intensive care unit (ICU) admission rates (*p* = 0.063), and higher GBS disability scores during peak illness (*p* = 0.048). The COVID-GBS group contained more patients with the highest GBS disability scores (5–6) than did the control group, including one fatality. Serum IL-6 was available in a subset of patients (COVID-GBS: *n* = 12; Non–COVID-GBS: *n* = 6). Our data revealed significantly elevated interleukin (IL)−6 levels in COVID-GBS patients compared to Non-COVID-GBS cases [median (IQR): 46.25 (11.78–89.24) vs. 7.05 (4.14–13.87) pg/mL, *p* = 0.032]. Furthermore, IL-6 levels were elevated in COVID-ICU patients compared to non-ICU patients [median (range): 50.95 (46–224) vs. 29.8 (5.19–40) pg/mL, *p* = 0.028], with both comparisons demonstrating statistical significance (Fig. 3).

**Table 1:** Demographic, clinical manifestations, laboratory findings, electrodiagnosis, treatment, disease progression, and prognosis of COVID-19-positive GBS versus COVID-19-negative patients.

Characteristic	Cases Group (COVID-GBS)	Control Group (Non-COVID-GBS)	Test statistic	*P* value
	(*n* = 55)	(*n* = 48)		
Male/Female	40/15	29/19	χ²= 1.757	0.185
Onset age (years)	54.04 ± 15.803	42.06 ± 21.147	**t = 3.22**	**0.002**
Signs and symptoms, n (%)				
Limb weakness	53/55 (96.4%)	45/48(93.8%)	-	0.662
Sensory disturbances	30/55 (54.5%)	24/48(50%)	χ²= 0.212	0.645
Pain	5/55 (9.1%)	3/48 (6.3%)	-	0.721
Areflexia or Hyporeflexia	50/55 (90.9%)	43/48 (89.6%)	χ²= 0.051	0.821
Cranial nerve involvement	24/55 (43.6%)	10/48 (20.8%)	**χ² = 6.027**	**0.014**
Oculomotor	13/55 (23.6%)	5/48 (10.4%)	χ²= 3.106	0.078
Facial	15/55 (27.3%)	7/48 (14.6%)	χ²= 2.457	0.117
Bulbar	11/55 (20%)	2/48 (4.2%)	**χ²= 6.745**	**0.009**
Clinical Subtypes			-	0.556
Classic GBS	50/55 (90.90%)	44/48 (91.66%)	-	1
MFS	3/55 (5.45%)	3/48 (6.25%)	-	1
Pharyngeal-cervical-brachial	2/55 (3.64%)	0	-	0.501
Pure sensory form	0	1/48 (2.08%)	-	0.466
ICU admission	15/55(27.3%)	6/48(12.5%)	χ² = 3.446	0.063
Ventilator dependency	12/55(21.8%)	4/48(8.3%)	χ² = 3.552	0.059
GBS disability score at nadir, (IQR)	3(3.0, 4.00)	3(2.0, 4.0)	**Z= −1.98**	**0.048**
Highest GBS disability score (%)				
0–2	10/55 (18.2%)	16(33.3%)	χ²= 3.118	0.077
3–4	33/55 (60%)	28(58.3%)	χ²= 0.029	0.864
5–6	12/55 (21.8%)	4(8.3%)	χ²= 3.552	0.059
GBS disability score at 6 months follow-up after discharge	1(1.0, 2.0)	1(1.0,2.0)	Z= −1.349	0.178
CSF				
albuminocytological dissociation	41/52(78.85%)	31/47(65.96%)	χ²=2.068	0.150
CSF protein concentrations, (mg/L)(IQR)	821.10 (529.30, 1232.58)	595.00 (394.20, 1082.60)	Z= −1.81	0.070
Leucocyte count (/μL) (IQR)	2 (2.0, 4.0)	2 (2.0, 2.0)	u= −0.195	0.845
Anti-ganglioside antibodies in serum or CSF	10/34	22/37	**χ²=6.462**	**0.011**
Electrodiagnosis			χ²=2.960	0.399
AIDP	29/48 (60.4%)	20/44(45.5%)	χ²= 2.064	0.151
AMAN	10/48 (20.8%)	16/44 (36.4%)	χ²= 2.731	0.098
AMSAN	6/48 (12.5%)	5/44 (11.4%)	χ²= 0.028	0.867
Equivocal	3/48 (6.3%)	3/44 (6.3%)	-	1
Treatment				
Steroids	0	1	NA	NA
IVIG	36	33	NA	NA
Plasma exchange	3	2	NA	NA
IVIG and Steroids	4	5	NA	NA
Steroids and Plasma exchange	2	1	NA	NA
IVIG, Steroids and Plasma exchange	2	2	NA	NA
IVIG and Plasma exchange	6	2	NA	NA
No treatment	2	2	NA	NA

Abbreviations: GBS = Guillain-Barré syndrome; CSF= cerebrospinal fluid; albumin-cytological dissociation=cell count < 50 cell/µL with elevated CSF proteins; AIDP= acute inflammatory demyelinating polyneuropathy; AMAN= acute motor axonal neuropathy; AMSAN= acute motor sensory axonal neuropathy; MFS = Miller-Fisher syndrome; IVIg = intravenous immunoglobulin; NA = not applicable.

Notes: Fisher’s Exact Test does not generate a test statistic; “-” is used in the table. The associated p-value is reported.

In the COVID-GBS group, 52 patients underwent lumbar puncture, with 41 exhibiting albuminocytological dissociation. In the Non-COVID-GBS group, 47 patients underwent the same procedure, with 31 demonstrating albuminocytological dissociation(*p* = 0.150). No statistically significant differences in protein or cellular levels were observed between groups. Antibodies against gangliosides (GM1, GM2, GM3, GM4, GD1a, GD1b, GD2, GD3, GT1a, GT1b, GQ1b) and sulfatides were detected in serum and/or CSF samples. In the COVID-GBS group, 10 patients (29.4%) tested positive for one or more anti-ganglioside antibodies. Among these seropositive patients, 7 exhibited a single antibody specificity, while 3 demonstrated multiple antibody specificities. In the Non-COVID GBS control group (*n* = 37), 22 patients (59.5%) tested positive for anti-ganglioside antibodies. The seropositivity rate in this control group was significantly higher than that in the COVID-GBS group (*p* = 0.011). Among the antibody-positive patients in the control group, 10 had a single antibody specificity and 12 had multiple specificities. The detailed distribution of specific antibody reactivities is presented in Table 2. Electrophysiological examinations were performed in 51 COVID-GBS patients, with raw data obtained from 48. In the Non-COVID-GBS group, 46 patients underwent electrophysiological examinations, with raw data obtained from 44. No significant differences in subtype distribution were observed between groups. The AIDP subtype predominated in COVID-GBS patients (29/48), while the AMAN subtype was less prevalent (10/48).

**Table 2:** Gangliosides Antibodies Spectrum in COVID-19-associated and Non-COVID-19 Guillain-Barré syndrome patients.​.

Positive Antibodies	Cases Group (COVID-GBS)	Control Group (Non-COVID-GBS)
	10/34 (29.41%)	22/37 (59.46%)
Anti-GM1-IgG	2/34 (5.88%)	7/37 (18.92%)
Anti-GM1-IgM	2/34 (5.88%)	1/37 (2.70%)
Anti-GM2-IgG	-	1/37 (2.70%)
Anti-GM2-IgM	-	1/37 (2.70%)
GM3-IgM	1/34 (2.94%)	1/37 (2.70%)
Anti-GD1a-IgG	-	3/37 (8.11%)
Anti-GD1b-IgG	-	4/37 (10.81%)
Anti-GD1b-IgM	-	1/37 (2.70%)
Anti-GD2-IgG	1/34 (2.94%)	-
Anti-GD3-IgG	-	1/37 (2.70%)
Anti-GD3-IgM	-	1/37 (2.70%)
Anti-GT1a-IgG	2/34 (5.88%)	4/37 (10.81%)
Anti-GT1a-IgM	-	1/37 (2.70%)
Anti-GQ1b-IgG	4/34 (11.76%)	1/37 (2.70%)
Anti- GQ1b-IgM	-	4/37 (10.81%)
Anti-Sulfatides-IgG	2/34 (5.88%)	5/37 (13.51%)
Anti-Sulfatides-IgM	-	3/37 (8.11%)

Notes: Data are presented as number of positive patients/total number of patients tested (n/N).

Nearly all patients received immunomodulatory treatment (COVID-GBS group: 53/55; Non-COVID-GBS group: 46/48). In the COVID-GBS group, treatment regimens included intravenous immunoglobulin (IVIG) alone (36, 65.5%), plasma exchange alone (3, 5.4%), IVIG plus steroids (4, 7.3%), steroids plus plasma exchange (2, 3.6%), IVIG plus steroids plus plasma exchange (2, 3.6%), and IVIG plus plasma exchange (6, 10.9%). In the Non-COVID-GBS group, treatments comprised steroids alone (1, 2.1%), IVIG alone (33, 68.8%), plasma exchange alone (2, 4.17%), IVIG plus steroids (5, 10.4%), steroids plus plasma exchange (1, 2.1%), IVIG plus steroids plus plasma exchange (2, 4.17%), and IVIG plus plasma exchange (2, 4.17%).

Post-discharge follow-up was conducted at 6 months via telephone, with patient outcomes assessed using the GBS disability score. Of the 103 patients initially enrolled, 92 (89.3%) completed the 6-month assessment, comprising 49 out of 55 (89.1%) in the COVID-19-positive (Omicron) group and 43 out of 48 (89.6%) in the COVID-19-negative group. No significant differences in prognostic outcomes were observed between groups based on 6-month post-discharge GBS disability scores. To investigate whether COVID-19-associated GBS is an independent factor influencing long-term prognosis, we performed a multivariable linear regression analysis. The 6-month GBS disability score was used as the dependent variable, with COVID-19 infection, age, sex, and the baseline GBS disability score as independent variables. The results showed that the model was statistically significant (F = 6.912, *p* < 0.001), with an adjusted R² of 0.206. After adjusting for other variables, Omicron infection status was not significantly associated with the 6-month prognosis (β = 0.042, 95% CI: −0.273 to 0.356, *p* = 0.793). The only factor independently associated with a poorer prognosis was a higher baseline GBS disability score (β = 0.282, 95% CI: 0.162 to 0.402, *p* < 0.001). This indicates that long-term prognosis differences are primarily driven by baseline factors rather than Omicron infection itself.

## Discussion

On December 7, 2022, China implemented the Notice on Further Optimizing the COVID-19 Prevention and Control Measures^21^. Following this policy adjustment, a rapid and widespread nationwide Omicron wave occurred, with sharply increased transmission and population-level exposure. This surge constituted a major shift in the epidemiological context and created a period of exceptionally high infection pressure. Thus, the period beginning in December 2022 was not only a critical turning point in the pandemic response strategy, but also marks the beginning of the national Omicron wave. This clear demarcation of phases provides a near “natural experiment” setting for our study to investigate the temporal association between GBS onset and SARS-CoV-2 infection under conditions of exceptionally high infection pressure. This two-center retrospective observational study, conducted at two hospitals, one is the largest comprehensive tertiary hospital in Henan Province, and the other is the largest tertiary medical center in Luoyang city, covering Henan Province, especially the two major cities of Zhengzhou and Luoyang, serving a population of more than 100 million, and being the main referral center for neurological emergency, to explore the potential relationship between COVID-19 and GBS.

During the 3-month period from December 2022 to February 2023, we observed a substantial increase in GBS cases compared to previous years, coinciding with the COVID-19 pandemic. Previous investigations examining the COVID-19-GBS relationship, primarily through comparing pre-pandemic and pandemic GBS incidence, reached similar conclusions. A retrospective multicenter study in two Italian SARS-CoV-2 hotspot regions documented increased GBS incidence in March and April 2020, with 30 patients compared to 17 during the same period in 2019^13^. A Spanish study encompassing 61 emergency departments^14^ identified a five-fold increase in GBS incidence during the COVID-19 pandemic peak (March-April 2020). The authors concluded that the relative GBS frequency was significantly higher in COVID-19 patients than in Non-COVID-19 patients^14^. However, some investigations of the COVID-19–GBS relationship have yielded conflicting results. Keddie et al.^16^ documented decreased GBS incidence during the UK COVID-19 pandemic, finding no epidemiological or phenotypic evidence supporting SARS-CoV-2 as a causative for GBS. The authors attributed this decline to lockdown implementation measures reducing transmission of GBS-associated pathogens such as *C. jejuni* and respiratory viruses. Similarly, Hafsteinsdottir et al.^22^ in Sweden found that the incidence of GBS decreased during the COVID-19 pandemic. Censi et al.^23^ conducted a systematic review and meta-analysis to discuss this debated issue and the observed decrease in the incidence of GBS during the pandemic may be attributed to health measures, which likely reduced the prevalence of infections that could have triggered this condition due to social restrictions. While these measures curbed SARS-CoV-2 transmission, they also drastically reduced the circulation of other common antecedent infections for GBS, such as *C. jejuni* and other respiratory viruses. Our study provides a unique counterpoint. It was conducted during China’s major COVID-19 outbreak beginning in December 2022, which occurred in the absence of social restrictions. This created a high-intensity, natural exposure context. The highly transmissible Omicron variant achieved extreme population penetration (estimated infection rate > 80%^3^ within a short period, unimpeded by lockdowns, thus minimizing the confounding “co-suppression” of other pathogens. Furthermore, a fully functional healthcare system ensured consistent case ascertainment, and the use of historical controls from the same institution mitigated biases from changing diagnostic practices. This unique context allowed us to observe the effects of SARS-CoV-2 more clearly. GBS has been reported to show seasonal variation, although the pattern appears to be region-dependent and may reflect seasonal changes in antecedent respiratory or gastrointestinal infections. A systematic review and meta-analysis^24^ reported an overall higher incidence in winter compared with summer, with substantial heterogeneity across regions and stronger seasonality among cases with a preceding respiratory illness. A national population-based study from China reported that GBS incidence was mostly concentrated in summer and autumn (*p* < 0.05), particularly in southeastern coastal areas^25^. In our study, incidence was assessed by comparing the same calendar period (December–February), and temporal factors were further adjusted using Poisson regression to minimize potential bias in between-group comparisons.

Weekly analysis of disease onset revealed that the incidence of GBS peaked between January 4 and January 10, 2023 (Fig. 2), occurring approximately 2 weeks after the COVID-19 peak in the region, consistent with the typical incubation period for an immune-mediated disease after infection. This timeline aligned with official surveillance data from the China CDC^26^, which documented: (1) clinic visits peaking at 2.867 million on December 23, 2022, followed by continuous decline; (2) nationwide SARS-CoV-2 nucleic acid test positivity peaking in December 17–20, 2022; and (3) highest nucleic acid test positivity rate (12.1%) occurring December 24–26, 2022.

Patients with COVID-19-associated GBS tended to be older, consistent with previous studies^27,28^, suggesting age-related susceptibility to COVID-19-associated GBS. This susceptibility may reflect age-related decline in immunomodulatory capacity, predisposing older individuals to immune response dysregulation following SARS-CoV-2 infection. Older individuals exhibit a chronic, low-grade pro-inflammatory state known as inflammaging^29^, characterized by elevated baseline levels of cytokines. Upon SARS-CoV-2 infection, this predisposes them to an exaggerated immune response, or a “cytokine storm”. This intense inflammatory response can disrupt the blood-nerve barrier, facilitating the entry of pathogenic antibodies and immune cells into the peripheral nervous system, thereby amplifying neural damage. Another significant clinical characteristic in the COVID-GBS group was a higher frequency of cranial nerve involvement. This could be attributed to the recognized neurotropic potential of SARS-CoV-2^30–32^ and the systemic inflammatory response it triggers. The associated “cytokine storm”, characterized by elevated IL-6 and IL-1β, may disrupt the blood-nerve barrier, potentially facilitating aberrant immune responses against peripheral nerves^33^. Moreover, higher cranial nerve involvement was found to be common in virus-associated GBS cases^11,34^. Studies^31,32^ demonstrate that coronaviruses possess neuroinvasiveness, neurotropism, and neurovirulence, consistent with the increased cranial nerve involvement frequency in COVID-19-associated GBS. The proportion of ganglioside antibody-positive patients was higher in the Non-COVID-GBS group. In the COVID-19-associated GBS group, the antibody response was significantly more restricted, with a majority of seropositive patients (7/10) exhibiting a single antibody specificity. In contrast, the non-COVID-19 GBS group displayed a more heterogeneous and broad immunoreactivity, with a majority of seropositive patients (12/22) showing multiple antibody specificities. This pattern suggests that the initial antigenic trigger may shape the subsequent autoimmune landscape. The broader antibody repertoire in the non-COVID-19 group is consistent with the classic understanding of GBS, which is often triggered by a variety of pathogens, each possessing distinct antigens that can induce a wide array of cross-reactive immune responses against peripheral nerve components. This finding may reflect methodological differences in antibody panel selection, varying antibody profiles, or distinct autoimmune pathogenesis between groups.

Patients with COVID-19-related GBS exhibited trends toward greater disease severity than did non-COVID-19 patients, including higher ventilator dependency rates, increased ICU admission rates, elevated GBS disability scores during peak illness (*p* = 0.048); additionally, they presented one case of death. These trends suggest potentially more severe clinical presentations in COVID-19-related GBS. Although rapidly progressive limb weakness and respiratory muscle paralysis due to GBS are usually the initial and main reasons for patients to be admitted to the ICU, we cannot rule out that in some patients, the disease may be aggravated by severe COVID-19 pneumonia or directly lead to respiratory failure. The requirement for mechanical ventilation in our cohort was primarily attributed to respiratory muscle weakness resulting from severe GBS, which was the initial indication for ventilator initiation in all cases. However, the clinical course of patients requiring prolonged ventilatory support was often complicated by secondary pulmonary infections. These complications, including pneumonitis, ventilator-associated pneumonia and/or aspiration pneumonia due to bulbar dysfunction, likely contributed to the extended duration of mechanical ventilation and complicated the weaning process in a subset of patients. Previous studies^13,14,35^ have documented increased severity of COVID-related GBS. Sharma et al.^35^ analyzed 13,705 GBS admissions in the U.S. during 2020, identifying 1,155 (8.43%) COVID-19-associated cases and required more invasive mechanical ventilation (20.8% vs. 11.8%, *p* < 0.001). Previously published data indicate increased ICU and mechanical ventilation requirements in patients with COVID-19-related GBS patients^13,36^. The limited statistical significance in some of our findings may reflect the predominant Omicron variant^1^ being less virulent, according to China CDC data^1^. Additionally, as most previous COVID-related GBS case reports originated from other countries, racial factors may contribute to observed differences.

Electrophysiological analysis revealed AIDP as the predominant COVID-GBS subtype, contrasting with the historical AMAN predominance^37,38^ in Chinese GBS populations. This finding aligns with AIDP being the major GBS subtype following influenza^39^ and other viruses^40^. Consistent with traditional AIDP patterns, patients with COVID-19-related GBS demonstrated higher cranial nerve involvement rates.

The treatment patterns were largely similar between the two groups, with most patients receiving IVIG as first-line therapy. The lack of significant differences in 6 months of GBS disability score outcomes between the two groups, as assessed by GBS disability scores, is reassuring and suggests that despite the initial severity of the disease, the long-term outcomes for both the case and control groups may be comparable. The lack of significant differences in treatment response is consistent with the findings of previous studies^13^. Our multivariate analysis demonstrates that Omicron infection status is not an independent predictor of the 6-month functional outcome​ (β = 0.042, *p* = 0.793). The primary determinant of a poorer long-term prognosis was unequivocally the severity of the neurological deficit at baseline, as measured by the GBS disability score (β = 0.282, *p* < 0.001). This suggests that while the virus may initiate the autoimmune response, it does not appear to exert a sustained, independent effect on the pace or extent of neurological recovery once the acute inflammatory phase has subsided. These results carry significant clinical implications. They should alleviate specific concerns regarding a uniquely severe prognosis for Omicron-associated GBS and refocus clinical efforts on aggressive management based on the initial disease severity. Early identification and intensive treatment of patients presenting with high baseline disability scores remain the paramount strategy for improving long-term outcomes, irrespective of the specific antecedent infection.

The occurrence of GBS linked to COVID-19, with a time gap between the two, indicates an immune-mediated mechanism rather than direct viral damage. Reports of COVID-19 complicated by GBS are common; however, the detection of SARS-CoV-2 RNA sequences in the CSF of patients with COVID-19-related GBS is rare. Therefore, researchers have speculated that the pathogenesis of COVID-19-related GBS more likely involves postinfectious immune responses than direct viral involvement. We performed a nucleotide BLAST (blastn) analysis. We compared the complete genomic sequences of the Omicron subvariants predominant in our study region and period (BA.5.2 and BF.7, GenBank accession numbers ​OR939743.1​ and ​OQ906631.1) against the human reference genome (taxid:9606) using the NCBI BLAST suite. No significant linear sequence homology was identified at the nucleotide level. This result aligns with the findings of a prior study^16^, which also reported a lack of significant homology between the ancestral SARS-CoV-2 genome and the human genome. However, the absence of linear genomic homology does not entirely preclude the possibility of molecular mimicry occurring at the protein structural or conformational level. Current evidence suggests GBS pathogenesis involves structural similarity between pathogen components and peripheral nerve components. The body’s immune system recognizes the error, and immune cells and antibodies attack normal peripheral nerve tissue, leading to peripheral nerve demyelination. Coronavirus attachment to respiratory epithelial cells is mediated by the spike (S) viral protein^41^, which utilizes the ACE-2 receptor for entry along with sialic acids linked to host cell surface gangliosides^42,43^. Dey et al.^44^ demonstrated that SARS-CoV-2 virions do not rely solely on the ACE2 receptor for cell entry. Using native-like lipid membranes, they showed the virus binds synergistically to both ACE2 and sialylated gangliosides, such as GD1a. Expanding on this, Negi et al.^43^ reported that evolved Variants of Concern—including Omicron, Delta, and B.1.1.8—specifically bind to terminal sialic acid residues on gangliosides such as GD1a, GM3, and GM1. Notably, the Omicron variant exhibits the highest binding affinity for GD1a, which contains two sialic acid residues. This reported binding of the SARS-CoV-2 spike protein to sialylated gangliosides supports the biological plausibility of a molecular mimicry pathway in GBS, as it indicates that the virus can interact with these glycolipids under experimental conditions. Taken together, these findings suggest a potential mechanistic contribution of SARS-CoV-2 infection to GBS pathogenesis. Furthermore, we observed that the level of IL-6 in patients with GBS associated with COVID-19 was higher than that in patients with Non-COVID-19 GBS. This may reflect COVID-19’s strong inflammatory response, producing increased IL-6 and other cytokines that exacerbate nervous system damage and potentially promote GBS development. Because IL-6 was not systematically measured in all participants, the observed IL-6 findings may be affected by selection bias and should be interpreted cautiously. Previous studies have documented increased cytokines, including IL-1β, IL-6, IL-17, tumor necrosis factor-α, and interferon-γ, plus various chemokines in patients with COVID-19^45^. As many of these cytokines are associated with the pathogenesis of typical GBS, the cytokine storm in COVID-19 may play a key role in the concurrent development of GBS^45^. Concurrently, inflammatory cytokines and the breakdown of the blood–nerve barrier^46^ exacerbate neural injury, amplifying clinical severity.

## Conclusion

Our study demonstrates a substantial increase in GBS incidence during the Omicron wave, with Poisson regression confirming a statistically significant 54% rise (IRR = 1.541, 95% CI 1.123–2.129, *p* = 0.0079). Patients with COVID-19-associated GBS were typically older and exhibited increased cranial nerve involvement frequency, particularly bulbar palsy, compared to those with Non-COVID-GBS. Although the COVID–GBS group demonstrated trends toward greater disease severity, long-term disability outcomes remained comparable between groups. These findings emphasize the necessity for heightened GBS vigilance in the post-COVID era, particularly among older adult patients and those with severe COVID-19. Further research is essential to elucidate underlying mechanisms and optimize management strategies for COVID-related GBS.

### Limitation

This study presents some limitations. First, this was a two-center retrospective study with a relatively small sample size, which may have led to sampling errors. A key limitation of this study is its reliance solely on hospitalized GBS cases. While the vast majority of GBS patients require hospitalization due to the severity of neurological deficits, our design likely missed the mildest cases that did not seek or require inpatient care. Consequently, we cannot calculate the absolute incidence of GBS within the Omicron-infected population. This was a retrospective study, and data collection relied on the clinical records of patients, with cases of incomplete or missing original data. The conclusions of this study are mainly based on clinical inferences rather than direct immunological evidence. Although we observed some trend differences in clinical features and disease severity in patients with COVID-19-associated GBS, we cannot rule out the influence of other potential factors or mechanisms owing to the lack of direct exploration of immunological mechanisms.

However, within this constraint, the study design offers unique strengths for assessing temporal association and healthcare burden. The two hospitals involved in this study are one is the largest tertiary medical center in Henan Province, and the other is the largest tertiary medical center in Luoyang city, covering Henan Province, especially the two major cities of Zhengzhou and Luoyang, serving a population exceeding 100 million. Therefore, the marked surge in GBS hospitalizations observed at these centers during the Omicron wave provides compelling evidence for a real increase in severe GBS cases requiring specialized care within the covered population.

## Data Availability

All data generated or analysed during this study are included in this published article.
